# Supplementary material for: A de novo strategy for predictive crystal engineering to tune excitonic coupling
Source: Nat Commun. 2019 May 3;10:2048. doi: 10.1038/s41467-019-10011-8 (PMC6499792; doi:10.1038/s41467-019-10011-8)
Supplement: Supplementary file 1 — Supplementary Information [file 41467_2019_10011_MOESM1_ESM.pdf]

Supplementary Information

A de novo strategy for predictive crystal engineering to tune excitonic coupling

Haldar *et al.*

Experimental section: materials and methods; synthesis of cNDI linkers

Materials:

Zinc acetate dihydrate, was purchased from Merck Millipore. Absolute ethanol was purchased from VWR (Germany).

Substrates:

The silicon substrates with a [100] orientation are from Silicon Sense (US). The quartz glasses are from Alfa Aesar. These substrates were treated with plasma (Diener Plasma) under O_2 (50 sccm) for 30 min to remove the impurities and generated a surface with hydroxyl groups.

Syntheses of the Me/Et/iPr-NDI(OEt)$_2$:

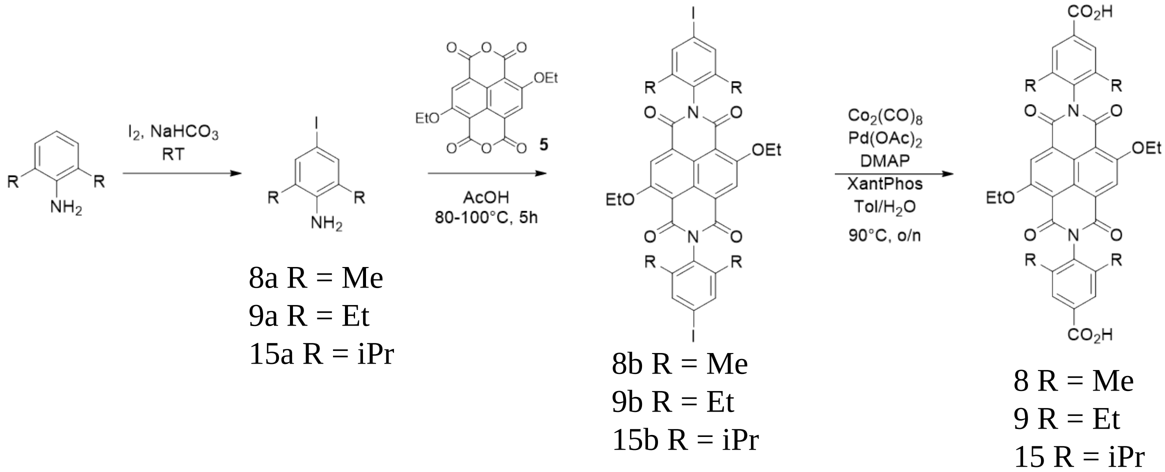

8a R = Me
9a R = Et
15a R = iPr

8b R = Me
9b R = Et
15b R = iPr

8 R = Me
9 R = Et
15 R = iPr

Supplementary information Figure 1**: MOF linker syntheses:** Synthesis scheme of the R-NDI(OEt)$_2$ linkers.

4-iodo-2,6-dimethylaniline (8a). 2,6-dimethylaniline (0.455 g, 3.75 mmol) and sodium bicarbonate (0.6 g, 7.14 mmol) were suspended in 20 mL of a water / methanol mixture (1/1). At 0 °C, iodine (1.0 g, 3.94 mmol, 1.05 eq) was added portion wise and the mixture was stirred at room temperature overnight. After extraction with dichloromethane, the organic phase was washed

with water and brine and rotary evaporated. The crude product was purified by column chromatography on SiO_2 with CH_2Cl_2 as eluent to afford **8a** (0.575 mg, 62 %) as a pale brownish solid. NMR (^1H, $CDCl_3$, 300 MHz) δ (ppm) 7.25 (s, 2H), 3.59 (b, 2H), 2.13 (s, 6H); NMR (^{13}C, $CDCl_3$, 75 MHz) δ (ppm) 142.5, 136.5, 124.1, 79.2, 17.2; HRMS (ASAP+) $[M+H]^+$ *m/z* 247.9945 found, 247.9936 calc.

4-iodo-2,6-diethylaniline (9a). 2,6-diethylaniline (0.588 g, 3.94 mmol) and sodium bicarbonate (0.6 g, 7.14 mmol) were suspended in 20 mL of a water / methanol mixture (1/1). At 0 °C, iodine (1.0 g, 3.94 mmol, 1. eq) was added portion wise and the mixture was stirred at room temperature overnight. After extraction with dichloromethane, the organic phase was washed with water and brine and rotary evaporated. The crude product was purified by column chromatography on SiO_2 with CH_2Cl_2 as eluent to afford **9a** (0.76 mg, 70 %) as a brownish oil. NMR (^1H, $CDCl_3$, 300 MHz) δ (ppm) 7.24 (s, 2H), 3.75 (b, 2H), 2.48 (q, 4H, J = 7.2 Hz) 1.24 (t, 6H, J = 7.0 Hz); NMR (^{13}C, $CDCl_3$, 75 MHz) δ (ppm) 141.3, 134.5, 130.3, 80.3, 24.0, 12.8; HRMS (ASAP+) $[M+H]^+$ *m/z* 276.0259 found, 276.0249 calc.

4-iodo-2,6-diisoproylaniline (15a). To a solution of 2,6-diisopropylaniline (4.7 g, 2.65 mmol) in diethyl ether (25 mL), iodine (7.4 g, 29.16 mmol) was added followed by 75 mL of an aqueous saturated solution of $NaHCO_3$. The mixture was stirred at room temperature for 3h. $Na_2S_2O_3$ was added until complete discoloration of the aqueous phase. The organic phase was recovered, washed with water and brine, dried onto $MgSO_4$ and concentrated in vacuo to afford **15a** as auburn oil (7.62 g, 95 %). NMR (^1H, $CDCl_3$, 300 MHz) δ (ppm) 7.29 (s, 2H), 3.74 (s, 2H), 2.85 (m, 2H), 1.25 (d, J = 6.8 Hz, 12H); NMR (^{13}C, $CDCl_3$, 75 MHz) δ (ppm) 140.12, 135.03, 131.74, 27.90, 22.28; TOF-MS (ES+) $[M+H]^+$ m/z 304.0562 found, 304.0562 calc.

8b. 4-iodo-2,6-dimethylaniline (**8a**) (241 mg, 0.975 mmol, 4 eq) and **4** (87 mg, 0.224 mmol, 1 eq) were suspended in acetic acid (10 mL) and heated at 80 °C for 5 hours. The mixture was then cooled down to room temperature and poured onto water. The aqueous phase was extracted 3 times with CH_2Cl_2. The organic phases were combined and washed with a saturated solution of $NaHCO_3$, water and brine, then dried onto $MgSO_4$ and concentrated under vacuum. Purification by column chromatography (SiO_2, CH_2Cl_2) afforded **8b** as a bright yellow powder (0.136 g, 75 %). NMR (^1H, $CDCl_3$, 300 MHz) δ (ppm) 8.57 (s, 2H), 7.59 (s, 4H), 4.50 (q, 4H, J = 7.0 Hz), 2.10 (s, 12H), 1.61 (t, 6H, J = 7.0 Hz); HRMS (ASAP-) $[M]^-$ *m/z* 814.0023 found, 814.0037 calc.

9b. 4-iodo-2,6-diethylaniline (**9a**) (309 mg, 1.12 mmol, 4 eq) and **4** (100 mg, 0.281 mmol, 1 eq) were suspended in acetic acid (10 mL) and heated at 80°C for 5 hours. The mixture was then cooled down to room temperature and poured onto water. The aqueous phase was extracted 3 times with CH_2Cl_2. The organic phases were combined and washed with a saturated solution of $NaHCO_3$, water and brine, then dried onto $MgSO_4$ and concentrated under vacuum. Purification by column chromatography (SiO_2, CH_2Cl_2) afforded **9b** as a bright yellow powder (75 mg, 31 %). NMR (^1H, CD_2Cl_2, 300 MHz) δ (ppm) 8.55 (s, 2H), 7.65 (s, 4H), 4.59 (q, 4H, J = 7.0 Hz), 2.39 (q, 8H, J = 7.0 Hz), 1.56 (t, 6H, J = 7.0 Hz), 1.11 (t, 12H, J = 7.0 Hz); HRMS (ASAP-) $[M]^-$ *m/z* 870.0645 found, 870.0663 calc.

15b. 4-iodo-2,6-diisoproylaniline (**15a**) (0.1 g, 0.281 mmol) was dissolved in a solution of **4** (0.350 g, 1.2 mmol, 4.1 eq) in acetic acid (10 mL). The resulting brown suspension was heated at 80°C for 5 hours. Upon heating the solution turned clear. The mixture was cooled down to room temperature and poured onto water. The aqueous phase was extracted 3 times with CH_2Cl_2. The organic phases were combined and washed with a saturated solution of $NaHCO_3$, water and brine, then dried onto $MgSO_4$ and concentrated under vacuum. Purification by column chromatography (SiO_2, PET/CH_2Cl_2, from 1:9 to 9:1) afforded **15b** as a bright yellow powder (61 mg, 23 %). NMR (^1H, CD_2Cl_2, 300 MHz) δ (ppm) 8.55 (s, 2H), 7.66 (s, 4H), 4.50 (q, J = 7.0 Hz, 4H), 2.65 (m, 4H), 1.55 (t, J = 7.0 Hz, 6H), 1.12 (dd, J_1 = 6.9 Hz, J_2 = 2.6 Hz, 24H); NMR (^{13}C, CD_2Cl_2, 75 MHz) δ (ppm) 162.82, 161.06, 148.88, 134.03, 131.58, 127.83, 124.95, 121.07, 96.19, 67.19, 29.48, 23.92, 23.85, 14.91; HRMS (ASAP+) $[M+H]^+$ *m/z* 927.1373 found, 927.1367 calc.

General procedure for carbopalladation reaction. In a sealable tube, **2**, palladium acetate (10 mol%), XantPhos (20 mol%) and DMAP (1 eq) were dissolved in toluene / water solution (3 to 1 ratio). $Co_2(CO)_8$ (0.33 eq) was added and the tube was sealed immediately. The resulting green mixture was heated at 90°C overnight under vigorous stirring. The mixture was cooled down to room temperature and the yellow suspension was poured onto water and the aqueous phase was extracted with ethyl acetate. The organic phase was washed with water (3x), citric acid (5% aq. 3x), water (1x) and brine (1x), dried onto $MgSO_4$ and concentrated in vacuo to afford a yellow powder. Washing with CH_2Cl_2 afforded the desired compound as a bright yellow powder.

Me-NDI(OEt)$_2$ (8). Following the general procedure, **8b** (60 mg, 0.074 mmol), palladium acetate (1.5 mg, 10 mol%), XantPhos (8 mg, 20 mol%) and DMAP (9 mg, 0.074 mmol, 1 eq) were dissolved in toluene / water solution (8 + 2 ml) and $Co_2(CO)_8$ (14 mg, 0.0409 mmol, 0.6 eq) was added. Washing with CH_2Cl_2 afforded the desired compound as a bright yellow powder (10 mg, 21 %). Due to solubility issues, only ^1H spectra were recorded. NMR (^1H, DMSO-d$_6$, 300 MHz) δ (ppm) 13.0 (b, 2H), 8.50 (s, 2H), 7.84 (s, 4H), 4.52 (q, 4H, *J* = 7.0 Hz), 2.12 (s, 12H), 1.44 (t, 6H, *J* = 7.0 Hz); HRMS (ASAP+) $[M+H]^+$ *m/z* 651.1982 found, 651.1979 calc.

Et-NDI(OEt)$_2$ (9). Following the general procedure, **9b** (64 mg, 0.074 mmol), palladium acetate (1.5 mg, 10 mol%), XantPhos (8 mg, 20 mol%) and DMAP (9 mg, 0.074 mmol, 1 eq) were dissolved in toluene / water solution (8 + 2 ml) and $Co_2(CO)_8$ (14 mg, 0.0409 mmol, 0.6 eq) was added. Washing with CH_2Cl_2 afforded the desired compound as a bright yellow powder (9 mg, 17 %). Due to solubility issues, only ^1H spectra were recorded. NMR (^1H, DMSO-d$_6$, 300 MHz) δ (ppm) 13.10 (b, 2H), 8.55 (s, 2H), 7.87 (s, 4H), 4.52 (q, 4H, *J* = 7.0 Hz), 2.46 (q, 8H, *J* = 7.1 Hz), 1.43 (t, 6H, *J* = 7.0 Hz), 1.08 (t, 12H, *J* = 7.0 Hz); HRMS (ASAP-) $[M]^-$ *m/z* 706.2529 found, 706.2526 calc.

iPr-NDI(OEt)$_2$ (15). Following the general procedure, **15b** (64 mg, 0.069 mmol), palladium acetate (1.52 mg, 10 mol%), XantPhos (7.8 mg, 20 mol%) and DMAP (8 mg, 0.069 mmol, 1 eq) were dissolved in toluene / water solution (8 + 2.5 mL). and $Co_2(CO)_8$ (7.1 mg, 0.0207 mmol, 0.33 eq) was added. Washing with CH_2Cl_2 afforded the desired compound as a bright yellow powder (45 mg, 85 %). Due to solubility issues, only ^1H spectra were recorded. NMR (^1H, THF-d8, 300 MHz) δ (ppm) 8.61 (s, 2H), 8.01 (s, 4H), 4.51 (q, 4H, *J* = 7.0 Hz), 2.81 (m, 4H), 1.50 (t, 6H, *J* = 6.98 Hz), 1.18 (m, 24H);

Supplementary Information Table 1: **Predicted dihedral and rotation angle for different "steric controls"**: The inter-molecular distances, dihedral angles (α1) and rotational angles (θ) calculated for R-NDI(OEt)$_2$ trimers with different R groups using molecular dynamics (MD) simulation (GROMACS-2018.4 package). Also see Figure 2b in the manuscript.

R	Inter-molecular distance (x Å)	Dihedral angle, α1 °	Rotation angle, *θ* °
H	5.8	69.1	41.7
1	7.8	82.5	11
2	5.4	85.7	13.5
3	7.0	88.2	28.1
4	6.6	88.8	28.5
5	6.4	89.2	30.3
6	6.2	87.6	34
7	7.4	88.6	35
8	6.6	89.7	40.9
9	6.6	87.7	42.5
10	7.6	86.2	42.7
11	8.0	86	42.9
12	7.2	87.3	45.4
13	8.0	59.5	59.3
14	8.0	59.8	60.3
15	6.8	87.4	62.7
16	8.0	87.1	67.3
17	7.8	83.8	68.4
18	8.0	59.4	71.1

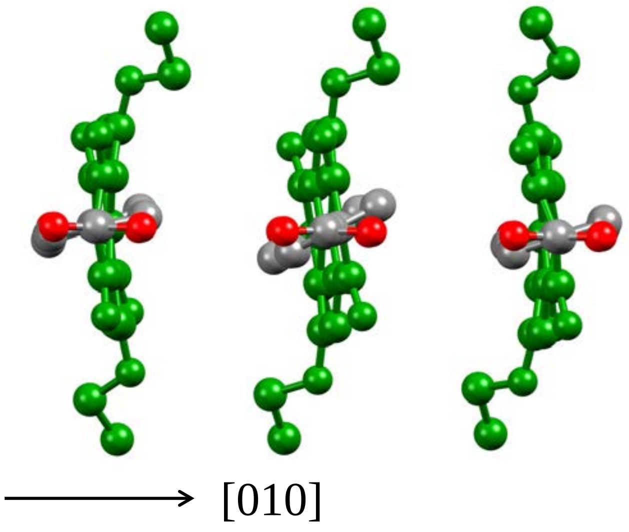

Supplementary Information Figure 2: **Cofacial Stacking of NDI(OEt)$_2$**: DFT Simulated geometry of the NDI(OEt)$_2$ in a trimer of NDI(OEt)$_2$ (a simplified model of Zn-(NDI(OEt)$_2$) (A) SURMOF) showing the co-facial stacking along [010] axis. NDI(OEt)$_2$ core is shown in green for clarity. Inter-NDI(OEt)$_2$ distance is 5.8 Å. The simulation method is described in the last part of the computational method in method section.

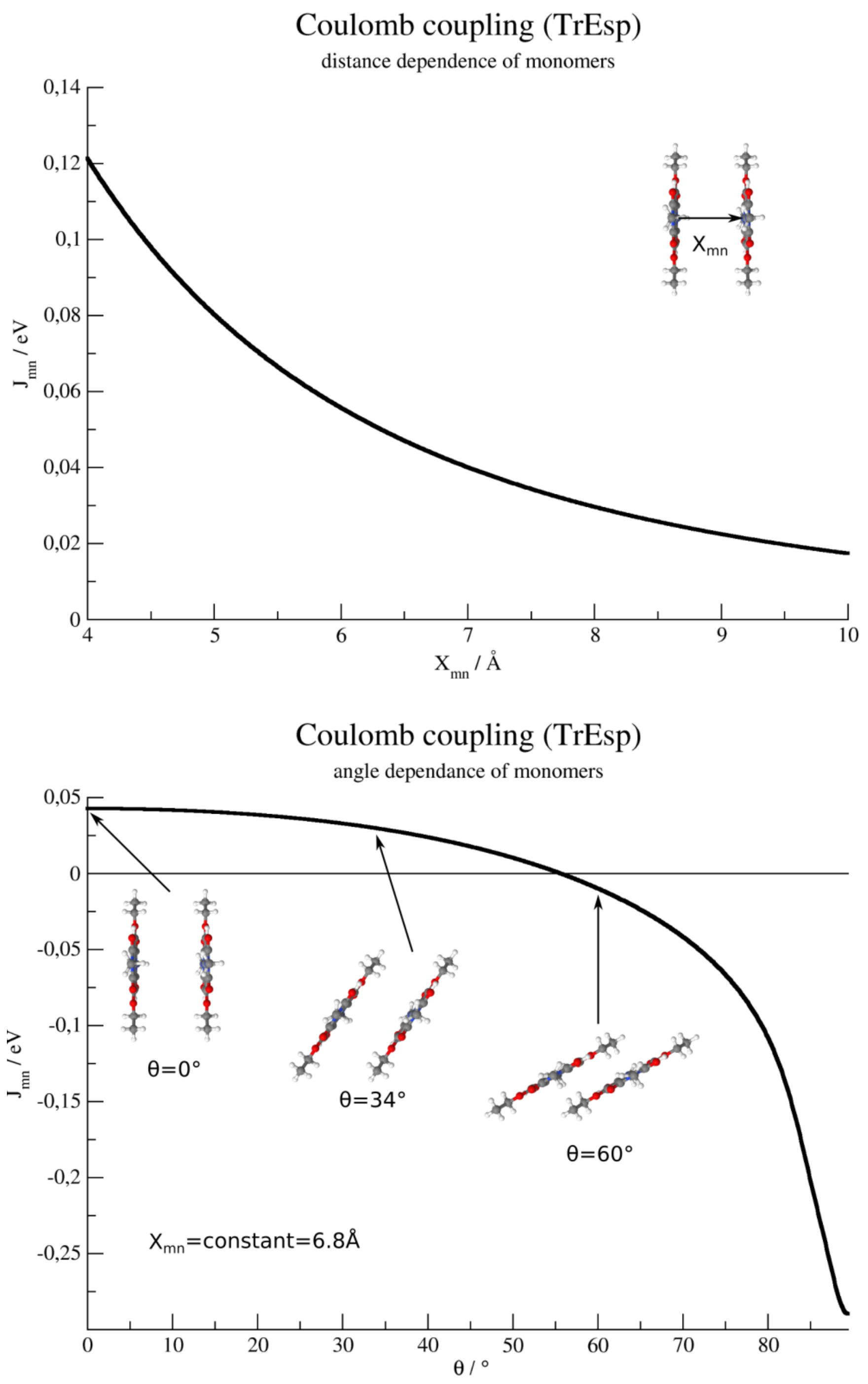

Supplementary Information Figure 3: **Coulomb coupling dependence on distance and angle**: Calculated Coulomb coupling between two NDI(OEt)$_2$ monomers: (top panel) distance dependence for rotation angle 0° and (bottom panel) angle θ dependence for fixed inter-NDI(OEt)$_2$ distance of 6.8 Å.

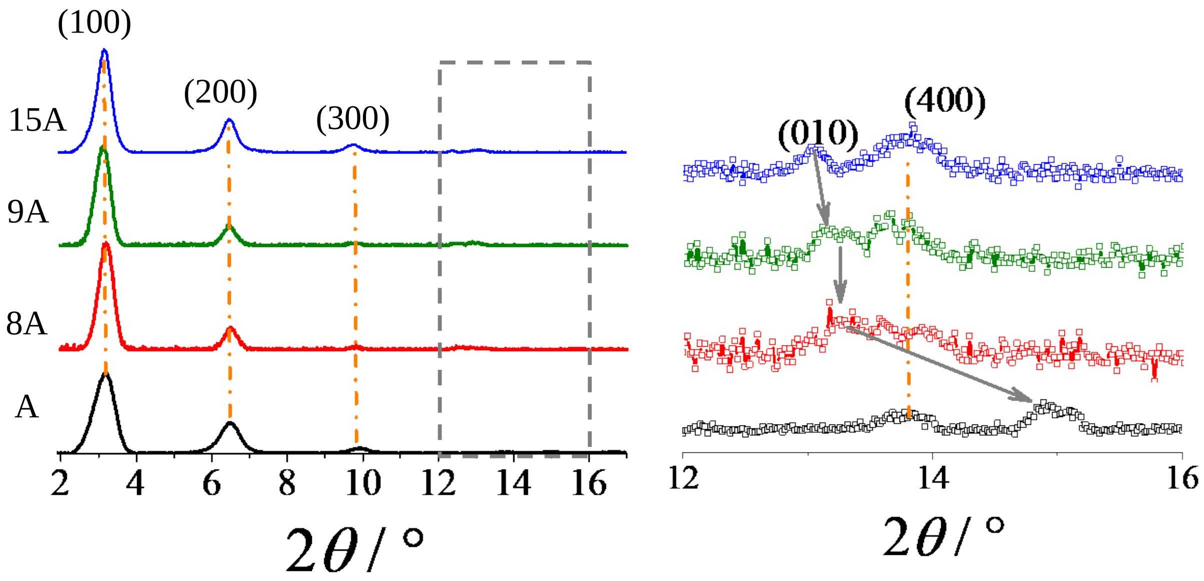

Supplementary Information Figure 4: **X-ray diffraction results**: In-plane XRD patterns of A, 8A, 9A and 15A, and the zoomed in patterns (right hand side) showing the (010) diffraction peaks. The shift in (010) diffraction from A to the other SURMOFs indicate an increase of ~1 Å in the inter-sheet distances.

Supplementary Information Table 2: **Rotation angles in optimized trimer structures:** The rotation angles are calculated (by DFT method) by considering a trimer of R-NDI(OEt)$_2$ linkers, having inter-chromophore distances as in the respective SURMOF-2 structures.

**SURMOFs**	**Simulated Rotation angle θ°**	**Inter-NDI distance Å**
**A**	1.82	5.8
**8A**	23.82	6.7
**9A**	28.07	6.7
**15A**	52.83	6.8

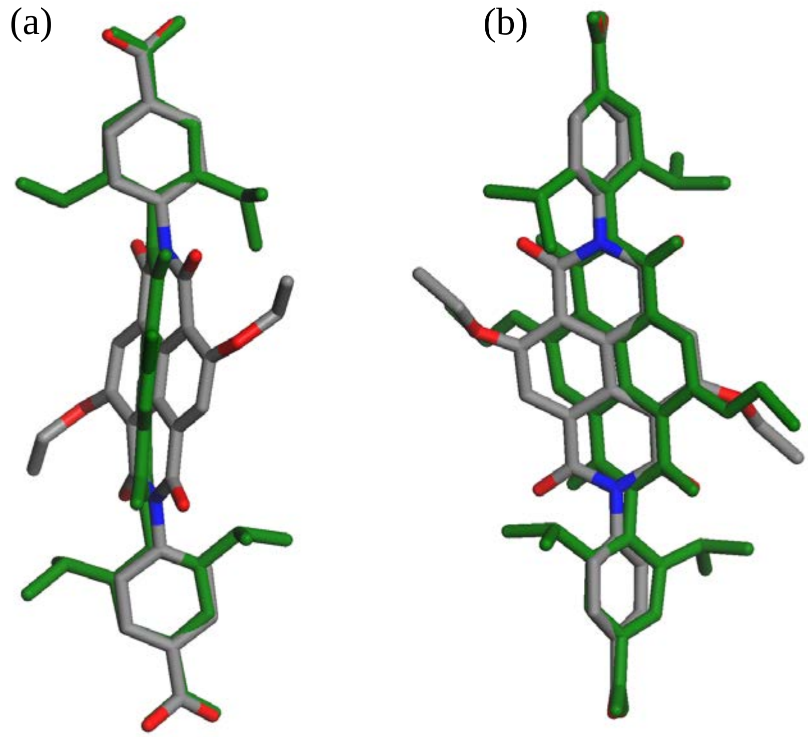

Supplementary information Figure 5: **Spatial geometry of central NDI(OEt)$_2$ in a trimer model of R=H and iPr**: DFT simulated geometries of central NDI(OEt)$_2$ showing the change of the dihedral angle (α1 and α2) due to the SCU. The green colored one is R=iPr, H-atoms are omitted for clarity, the carboxylate groups of two different NDI(OEt)$_2$ are superimposed to demonstrate the difference in spatial geometry. (a) and (b) are 90 ° rotated view, for a clear demonstration. In (b) the dihedral angle α2 is evident. It is important to note that, if there were no interactions between adjacent linkers, the dihedral angle α1 would be identical to the rotation angle θ (angle between cNDI plane and the carboxylate plane). Now, because of interactions between adjacent linkers, which are simulated by the GROMACS MD, α2 starts to deviate from 0 (i.e. there is a torsion between carboxylate plane and phenyl plane, as shown in figure 5b). As a result, for the MD optimized structure, the dihedral angle α1 and the rotational angle θ can be different.

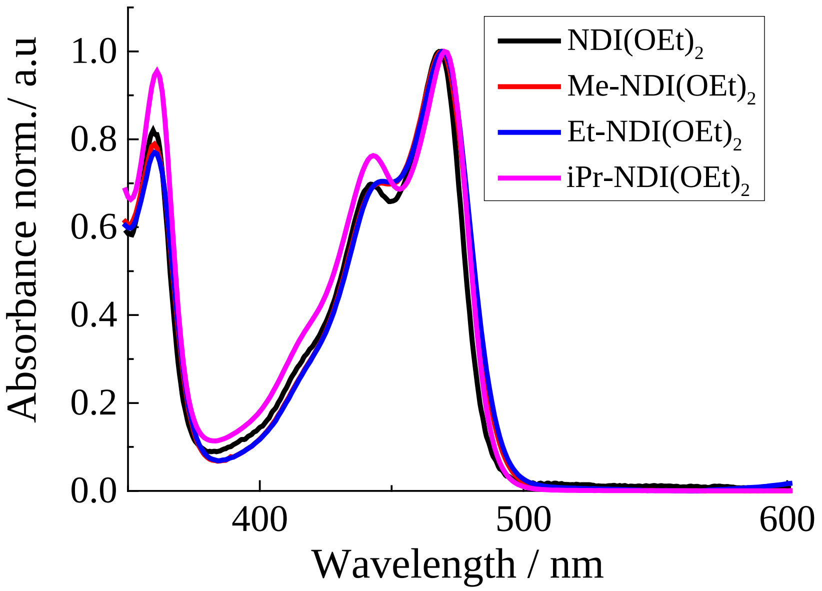

Supplementary information Figure 6: **UV-Vis spectra**: UV-Vis spectra of NDI(OEt)$_2$, Me-NDI(OEt)$_2$, Et-NDI(OEt)$_2$and iPr-NDI(OEt)$_2$ in ethanol (20 μM).

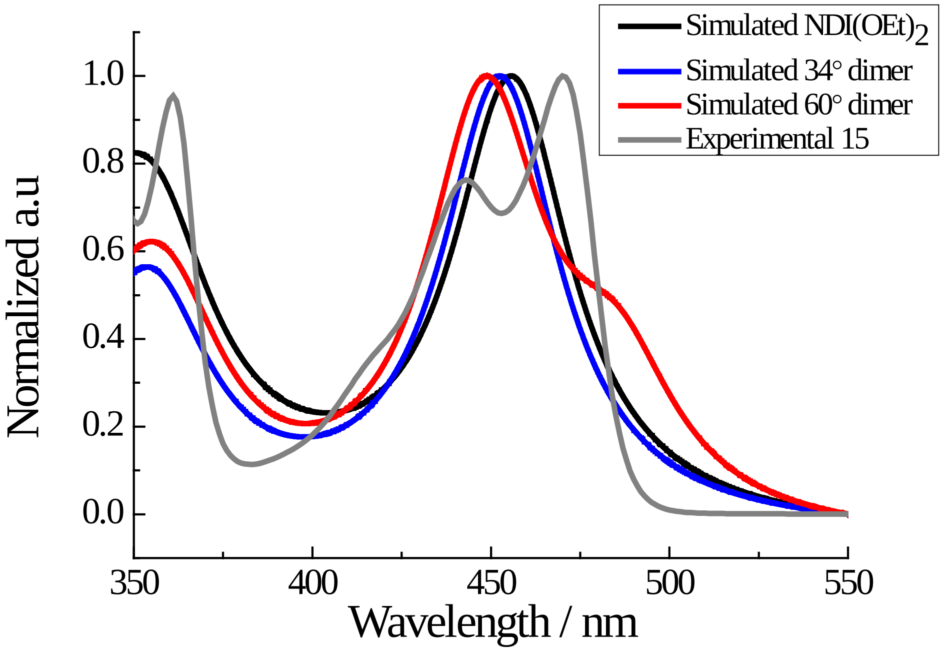

Supplementary Information Figure 7: **Simulated UV-Vis spectra**: DFT simulated electronic absorption spectra for NDI(OEt)$_2$ monomer, and dimers (inter-NDI(OEt)$_2$ distance ~ 6.8 Å) with rotation angle 34 and 60 °, and experimental absorption spectrum of iPr-NDI(OEt)$_2$ (15) in ethanol.

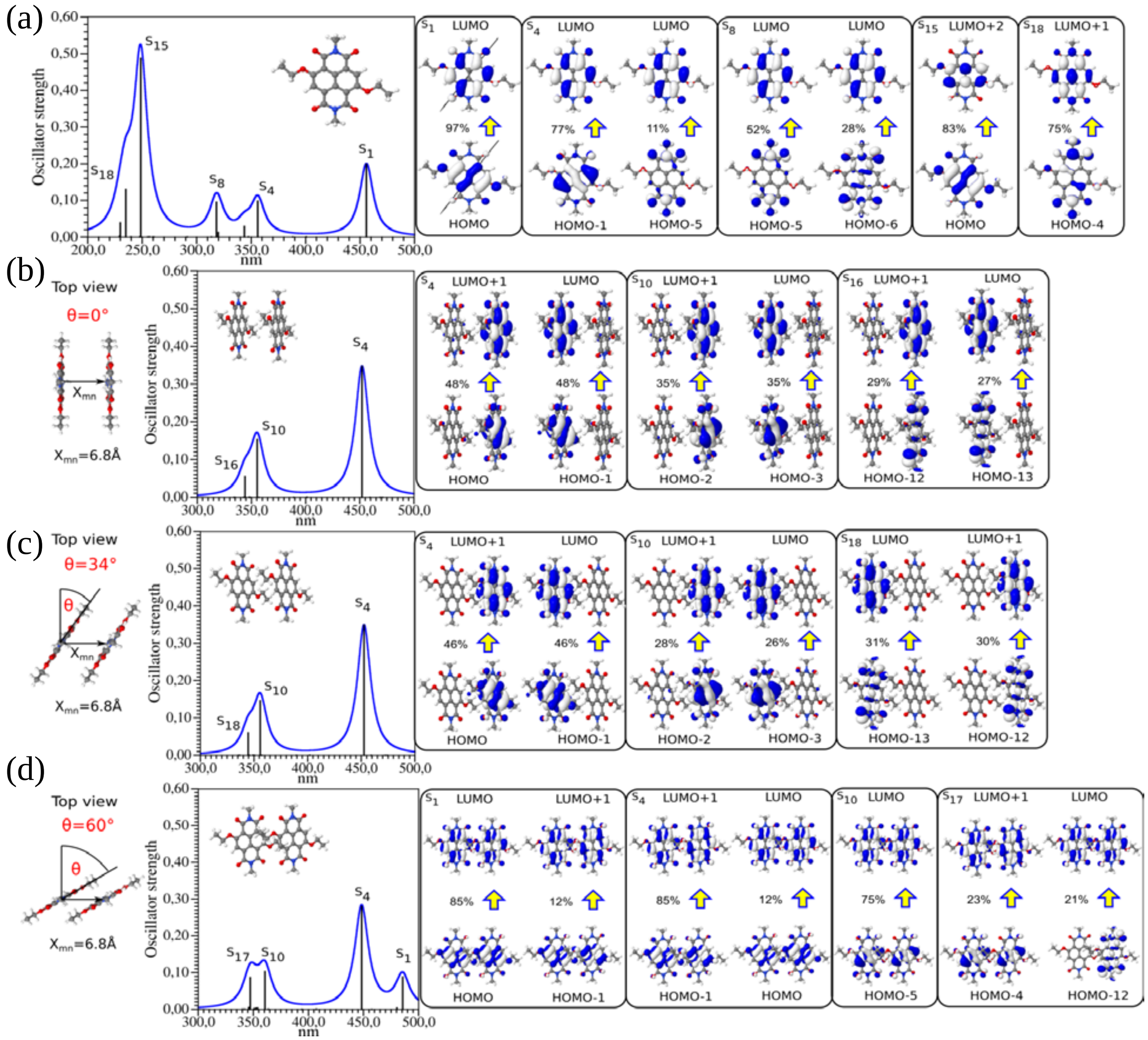

Supplementary information Figure 8: **Simulated electronic spectrum of the NDI(OEt)$_2$ monomer (a) and dimers (b-d)**: (left) TD-DFT simulated UV-Vis absorption spectrum for NDI(OEt)$_2$ dye dimers with rotation angles 0, 34 and 60° (the blue full line corresponds to Lorentzian broadening with half width of 15 nm, the black vertical lines correspond to values of oscillator strength f_e). (right) analysis of leading excitations contributing to the most intense transitions; cut-off for MOs is 0.03, minus and plus are labeled by blue and white colours, respectively.

Supplementary Information Table 3: “**Calculated singlet excitations”**: TD-DFT calculated electronic transition wavelengths (nm) for monomer and three different rotations (0°, 34° and 60°) of monomers in dimer of $NDI(OEt)_2$ core models. The most intensive transitions are shown in bold and are depicted in the Figure 8 together with corresponding transitions analyses.

	**monomer**	**dimer 0°**	**dimer 34°**	**dimer 60°**
S1	**455.86**	471.00	474.16	**485.62**
S2	400.55	468.79	472.27	480.32
S3	388.23	459.36	459.34	457.45
S4	**356.10**	**452.10**	**452.60**	**448.33**
S5	347.54	401.38	402.05	407.23
S6	343.80	401.36	402.05	406.98
S7	319.79	389.00	389.65	394.40
S8	**318.16**	388.96	389.59	393.86
S9	313.55	357.15	357.36	363.61
S10	291.44	**355.40**	**355.90**	**360.55**
S11	260.81	349.03	350.62	353.95
S12	259.64	348.10	349.57	353.70
S13	256.57	347.92	349.21	352.32
S14	254.91	347.85	349.11	351.49
S15	**248.61**	344.50	345.76	350.83
S16	247.65	**344.04**	345.24	350.62
S17	239.70	343.75	344.96	**347.27**
S18	**234.85**	342.66	**344.57**	345.75
S19	229.98	334.91	336.62	342.07
S20	229.13	333.93	335.49	340.16

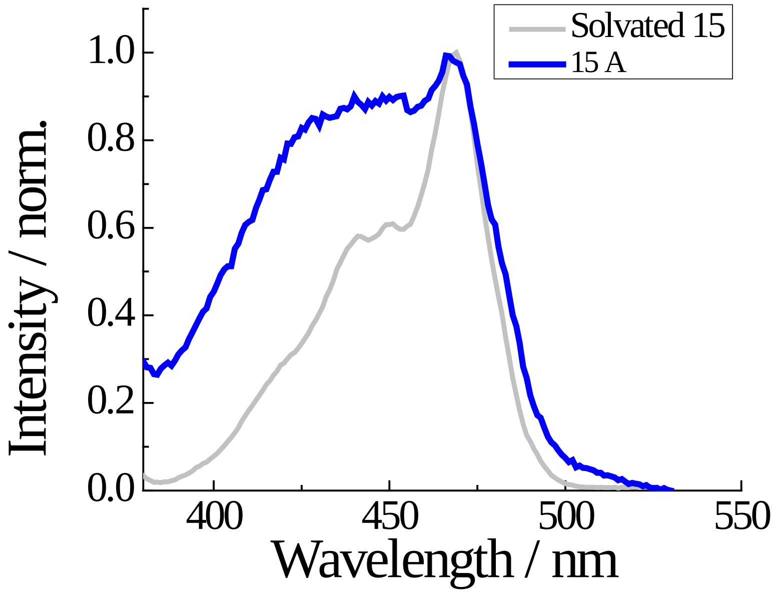

Supplementary Information Figure 9: **Excitation spectra**: Excitation spectra of 15A (blue) and solvated iPr-NDI(OEt)$_2$ (gray), monitored at 580 nm. The additional broadening of the excitation spectrum ~500 nm suggests that the emissive state is a J-aggregated state, but not a typical excimer.

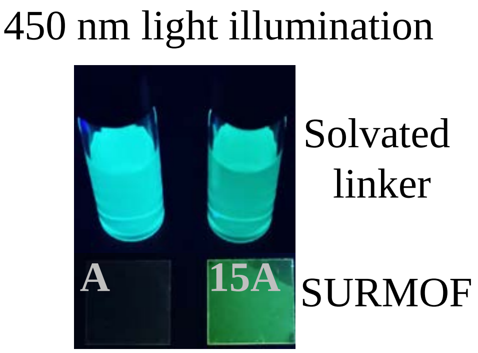

Supplementary Information Figure 10: **Fluorescence of monomer and respective SURMOFs**: Photograph of linker solutions and respective SURMOF thin films deposited on a quartz substrate under 450 nm light illumination.

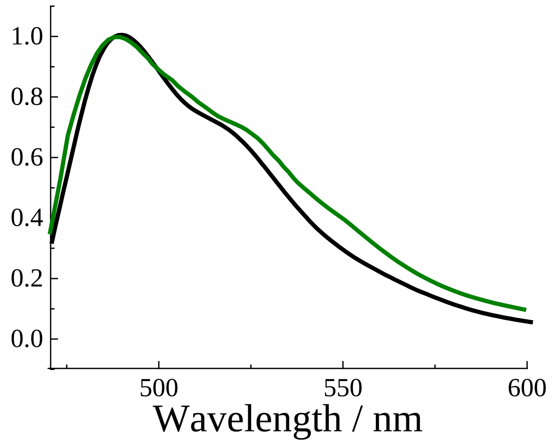

Supplementary Information Figure 11: **Fluorescence spectra of A and 15**: PL spectra of A (green) and 15 (black) recorded in ethanol (20 μM) upon excitation at 450 nm, recorded at room temperature.

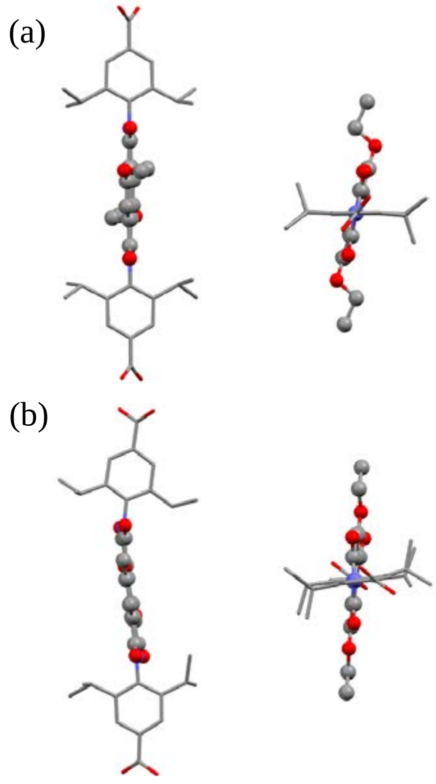

Supplementary Information Figure 12: **Comparative view of the linker (15) geometry**: (a) MD and (b) DFT optimized geometries.

Supplementary Information Table 3

TrEsp charges used to calculate Coulomb coupling

Atom type	Coordinates			Transition charge
C	-2.476500	0.003700	0.697100	-0.110102
C	-1.258300	-0.007400	1.431300	0.152483
C	-0.023500	0.000000	0.716900	-0.051436
C	0.021900	0.008100	-0.716600	0.053169
C	-1.218900	0.019200	-1.415000	-0.088180
C	-2.426100	0.020100	-0.724200	-0.024794
C	1.217500	-0.002900	1.413200	0.083144
C	1.266700	-0.004400	2.900900	-0.009351
N	0.026800	-0.002800	3.549700	-0.019605
C	-1.249500	-0.038000	2.919600	0.008187
C	1.258000	0.006200	-1.430200	-0.152738
C	2.476100	0.001000	-0.695400	0.109763
C	2.425100	-0.003500	0.725400	0.027563
C	-1.269700	0.032000	-2.903200	0.010920
N	-0.027300	0.022700	-3.549600	0.020118
C	1.247600	0.007700	-2.917900	-0.007039
O	-2.253600	-0.088400	3.620200	0.063036
O	2.325500	-0.005800	3.527000	0.040359
C	0.030900	-0.010700	5.012000	-0.002449
O	3.635900	0.000600	-1.368500	0.043038
C	4.885900	0.005900	-0.665400	-0.027700
C	5.989400	0.010800	-1.705200	-0.013356

O	-2.331500	0.047800	-3.525400	-0.040645
C	-0.008400	0.026300	-5.011700	0.000683
O	2.249000	-0.003400	-3.625100	-0.063881
O	-3.636900	0.001900	1.370300	-0.043090
C	-4.886700	-0.006000	0.666400	0.028400
C	-5.991500	-0.028800	1.704900	0.011800
H	3.337000	-0.009100	1.337200	-0.017187
H	-3.339500	0.032500	-1.335000	0.017011
H	-4.937200	-0.899500	0.003700	-0.014221
H	-4.958900	0.897600	0.019200	-0.013893
H	-6.981301	-0.031000	1.206800	-0.004686
H	-5.912600	-0.932400	2.340400	-0.011654
H	-5.929700	0.860500	2.361800	-0.011299
H	4.952300	-0.891900	-0.010600	0.013647
H	4.944600	0.904600	-0.010700	0.014011
H	6.979501	0.013200	-1.208700	0.005083
H	5.920100	-0.884500	-2.353000	0.012073
H	5.914600	0.906900	-2.350500	0.011683
H	-0.012500	-1.012800	-5.395100	-0.006180
H	-0.910400	0.561400	-5.354700	-0.006591
H	0.900900	0.538700	-5.380300	-0.004318
H	0.063700	-1.050100	5.399200	0.006143
H	0.923600	0.539000	5.355700	0.007275
H	-0.886700	0.481100	5.378500	0.004805

Supplementary Information Table 4

Structures Cartesian coordinates (in Angstroms)

NDI(OEt)$_2$ Dye monomer

C -2.4765 0.0037 0.6971

C -1.2583 -0.0074 1.4313

C -0.0235 -0.0 0.7169

C 0.0219 0.0081 -0.7166

C -1.2189 0.0192 -1.415

C -2.4261 0.0201 -0.7242

C 1.2175 -0.0029 1.4132

C 1.2667 -0.0044 2.9009

N 0.0268 -0.0028 3.5497

C -1.2495 -0.038 2.9196

C 1.258 0.0062 -1.4302

C 2.4761 0.001 -0.6954

C 2.4251 -0.0035 0.7254

C -1.2697 0.032 -2.9032

N -0.0273 0.0227 -3.5496

C 1.2476 0.0077 -2.9179

O -2.2536 -0.0884 3.6202

O 2.3255 -0.0058 3.527

C 0.0309 -0.0107 5.012

O 3.6359 0.0006 -1.3685

C 4.8859 0.0059 -0.6654

C 5.9894 0.0108 -1.7052

O -2.3315 0.0478 -3.5254

C -0.0084 0.0263 -5.0117

O 2.249 -0.0034 -3.6251

O -3.6369 0.0019 1.3703

C -4.8867 -0.006 0.6664

C -5.9915 -0.0288 1.7049

H 3.337 -0.0091 1.3372

H -3.3395 0.0325 -1.335

H -4.9372 -0.8995 0.0037

H -4.9589 0.8976 0.0192

H -6.9813 -0.031 1.2068

H -5.9126 -0.9324 2.3404

H -5.9297 0.8605 2.3618

H 4.9523 -0.8919 -0.0106

H 4.9446 0.9046 -0.0107

H 6.9795 0.0132 -1.2087

H 5.9201 -0.8845 -2.353

H 5.9146 0.9069 -2.3505

H -0.0125 -1.0128 -5.3951

H -0.9104 0.5614 -5.3547

H 0.9009 0.5387 -5.3803

H 0.0637 -1.0501 5.3992

H 0.9236 0.539 5.3557

H -0.8867 0.4811 5.3785

NDI(OEt)$_2$ Dye dimer 0° rotation

C -2.4765 0.0037 0.6971

C -2.4765 6.8037 0.6971

C -1.2583 -0.0074 1.4313

C -1.2583 6.7926 1.4313

C -0.0235 0.0 0.7169

C -0.0235 6.8 0.7169

C 0.0219 0.0081 -0.7166

C 0.0219 6.8081 -0.7166

C -1.2189 0.0192 -1.415

C -1.2189 6.8192 -1.415

C -2.4261 0.0201 -0.7242

C -2.4261 6.8201 -0.7242

C 1.2175 -0.0029 1.4132

C 1.2175 6.7971 1.4132

C 1.2667 -0.0044 2.9009

C 1.2667 6.7956 2.9009

N 0.0268 -0.0028 3.5497

N 0.0268 6.7972 3.5497

C -1.2495 -0.038 2.9196

C -1.2495 6.762 2.9196

C 1.258 0.0062 -1.4302

C 1.258 6.8062 -1.4302

C 2.4761 0.001 -0.6954

C 2.4761 6.801 -0.6954

C 2.4251 -0.0035 0.7254

C 2.4251 6.7965 0.7254

C -1.2697 0.032 -2.9032

C -1.2697 6.832 -2.9032

N -0.0273 0.0227 -3.5496

N -0.0273 6.8227 -3.5496

C 1.2476 0.0077 -2.9179

C 1.2476 6.8077 -2.9179

O -2.2536 -0.0884 3.6202

O -2.2536 6.7116 3.6202

O 2.3255 -0.0058 3.527

O 2.3255 6.7942 3.527

C 0.0309 -0.0107 5.012

C 0.0309 6.7893 5.012

O 3.6359 0.0006 -1.3685

O 3.6359 6.8006 -1.3685

C 4.8859 0.0059 -0.6654

C 4.8859 6.8059 -0.6654

C 5.9894 0.0108 -1.7052

C 5.9894 6.8108 -1.7052

O -2.3315 0.0478 -3.5254

O -2.3315 6.8478 -3.5254

C -0.0084 0.0263 -5.0117

C -0.0084 6.8263 -5.0117

O 2.249 -0.0034 -3.6251

O 2.249 6.7966 -3.6251

O -3.6369 0.0019 1.3703

O -3.6369 6.8019 1.3703

C -4.8867 -0.006 0.6664

C -4.8867 6.794 0.6664

C -5.9915 -0.0288 1.7049

C -5.9915 6.7712 1.7049

H 3.337 -0.0091 1.3372

H 3.337 6.7909 1.3372

H -3.3395 0.0325 -1.335

H -3.3395 6.8325 -1.335

H -4.9372 -0.8995 0.0037

H -4.9372 5.9005 0.0037

H -4.9589 0.8976 0.0192

H -4.9589 7.6976 0.0192

H -6.9813 -0.031 1.2068

H -6.9813 6.769 1.2068

H -5.9126 -0.9324 2.3404

H -5.9126 5.8676 2.3404

H -5.9297 0.8605 2.3618

H -5.9297 7.6605 2.3618

H 4.9523 -0.8919 -0.0106

H 4.9523 5.9081 -0.0106

H 4.9446 0.9046 -0.0107

H 4.9446 7.7046 -0.0107

H 6.9795 0.0132 -1.2087

H 6.9795 6.8132 -1.2087

H 5.9201 -0.8845 -2.353

H 5.9201 5.9155 -2.353

H 5.9146 0.9069 -2.3505

H 5.9146 7.7069 -2.3505

H -0.0125 -1.0128 -5.3951

H -0.0125 5.7872 -5.3951

H -0.9104 0.5614 -5.3547

H -0.9104 7.3614 -5.3547

H 0.9009 0.5387 -5.3803

H 0.9009 7.3387 -5.3803

H 0.0637 -1.0501 5.3992

H 0.0637 5.7499 5.3992

H 0.9236 0.539 5.3557

H 0.9236 7.339 5.3557

H -0.8867 0.4811 5.3785

H -0.8867 7.2811 5.3785

NDI(OEt)$_2$ Dye dimer 34° rotation

C -2.05104253469 1.38790866446 0.6971

C -2.05104253469 8.18790866446 0.6971

C -1.04731600503 0.6974975524 1.4313

C -1.04731600503 7.4974975524 1.4313

C -0.019482382955 0.0131410332316 0.7169

C -0.019482382955 6.81314103323 0.7169

C 0.0226853853571 -0.00553112024831 -0.7166

C 0.0226853853571 6.79446887975 -0.7166

C -0.999777393441 0.697517751434 -1.415

C -0.999777393441 7.49751775143 -1.415

C -2.00008827742 1.37332155832 -0.7242

C -2.00008827742 8.17332155832 -0.7242

C 1.00773158517 -0.683221568936 1.4132

C 1.00773158517 6.11677843106 1.4132

C 1.04768144438 -0.711977416146 2.9009

C 1.04768144438 6.08802258385 2.9009

N 0.0206524668148 -0.0173076750162 3.5497

N 0.0206524668148 6.78269232498 3.5497

C -1.05713177724 0.66720810513 2.9196

C -1.05713177724 7.46720810513 2.9196

C 1.04639626228 -0.698324639616 -1.4302

C 1.04639626228 6.10167536038 -1.4302

C 2.05333912631 -1.38378851071 -0.6954

C 2.05333912631 5.41621148929 -0.6954

C 2.00854184204 -1.35900034171 0.7254

C 2.00854184204 5.44099965829 0.7254

C -1.03473483296 0.736536431859 -2.9032

C -1.03473483296 7.53653643186 -2.9032

N -0.00993904682197 0.0340851191618 -3.5496

N -0.00993904682197 6.83408511916 -3.5496

C 1.03861306088 -0.691265477061 -2.9179

C 1.03861306088 6.10873452294 -2.9179

O -1.91775172618 1.18691020585 3.6202

O -1.91775172618 7.98691020585 3.6202

O 1.92468355614 -1.30521151494 3.527

O 1.92468355614 5.49478848506 3.527

C 0.0196338969248 -0.0261497627436 5.012

C 0.0196338969248 6.77385023726 5.012

O 3.01463322579 -2.03267205519 -1.3685

O 3.01463322579 4.76732794481 -1.3685

C 4.05389391388 -2.72726928539 -0.6654

C 4.05389391388 4.07273071461 -0.6654

C 4.97147692042 -3.34027637026 -1.7052

C 4.97147692042 3.45972362974 -1.7052

O -1.90617167963 1.34338625041 -3.5254

O -1.90617167963 8.14338625041 -3.5254

C 0.00774285775182 0.0265009085474 -5.0117

C 0.00774285775182 6.82650090855 -5.0117

O 1.8626042448 -1.26044356765 -3.6251

O 1.8626042448 5.53955643235 -3.6251

O -3.01406428111 2.03530384202 1.3703

O -3.01406428111 8.83530384202 1.3703

C -4.05461306323 2.72763373596 0.6664

C -4.05461306323 9.52763373596 0.6664

C -4.98328337158 3.32652799906 1.7049

C -4.98328337158 10.1265279991 1.7049

H 2.76140972419 -1.87357096079 1.3372

H 2.76140972419 4.92642903921 1.3372

H -2.75039720418 1.89436842225 -1.335

H -2.75039720418 8.69436842225 -1.335

H -4.59611831989 2.0151279065 0.0037

H -4.59611831989 8.8151279065 0.0037

H -3.60918286839 3.51712581415 0.0192

H -3.60918286839 10.3171258141 0.0192

H -5.80509498529 3.87819325225 1.2068

H -5.80509498529 10.6781932523 1.2068

H -5.42315901469 2.53328932841 2.3404

H -5.42315901469 9.33328932841 2.3404

H -4.43475860054 4.02923299089 2.3618

H -4.43475860054 10.8292329909 2.3618

H 3.60689861996 -3.50870962682 -0.0106

H 3.60689861996 3.29129037318 -0.0106

H 4.60510508174 -2.01503784237 -0.0107

H 4.60510508174 4.78496215763 -0.0107

H 5.79364908397 -3.89194357382 -1.2087

H 5.79364908397 2.90805642618 -1.2087

H 4.41337921016 -4.04376164076 -2.353

H 4.41337921016 2.75623835924 -2.353

H 5.41055767079 -2.55554817232 -2.3505

H 5.41055767079 4.24445182768 -2.3505

H -0.576713542292 -0.83265934219 -5.3951

H -0.576713542292 5.96734065781 -5.3951

H -0.440824910046 0.974510912552 -5.3547

H -0.440824910046 7.77451091255 -5.3547

H 1.04811716621 -0.0571743464014 -5.3803

H 1.04811716621 6.7428256536 -5.3803

H -0.534398774563 -0.906192942891 5.3992

H -0.534398774563 5.89380705711 5.3992

H 1.06710407698 -0.0696193140384 5.3557

H 1.06710407698 6.73038068596 5.3557

H -0.466079909725 0.894686323664 5.3785

H -0.466079909725 7.69468632366 5.3785

NDI(OEt)$_2$ Dye dimer 60° rotation

C -1.23504570601 2.14656191247 0.6971

C -1.23504570601 8.94656191247 0.6971

C -0.635558587988 1.08601976558 1.4313

C -0.635558587988 7.88601976558 1.4313

C -0.01175 0.0203515969889 0.7169

C -0.01175 6.82035159699 0.7169

C 0.0179648057707 -0.0149159563429 -0.7166

C 0.0179648057707 6.78508404366 -0.7166

C -0.592822312247 1.06519836467 -1.415

C -0.592822312247 7.86519836467 -1.415

C -1.19564288938 2.11111423212 -0.7242

C -1.19564288938 8.91111423212 -0.7242

C 0.606238526329 -1.05583592911 1.4132

C 0.606238526329 5.74416407089 1.4132

C 0.629539488223 -1.09919437897 2.9009

C 0.629539488223 5.70080562103 2.9009

N 0.0109751288694 -0.0246094808214 3.5497

N 0.0109751288694 6.77539051918 3.5497

C -0.657658965344 1.06309874203 2.9196

C -0.657658965344 7.86309874203 2.9196

C 0.634369357503 -1.08635995796 -1.4302

C 0.634369357503 5.71364004204 -1.4302

C 1.2389160254 -2.14386550231 -0.6954

C 1.2389160254 4.65613449769 -0.6954

C 1.20951891109 -2.10194820672 0.7254

C 1.20951891109 4.69805179328 0.7254

C -0.607137187079 1.11559245519 -2.9032

C -0.607137187079 7.91559245519 -2.9032

N 0.00600877666591 0.0349924935233 -3.5496

N 0.00600877666591 6.83499249352 -3.5496

C 0.630468395609 -1.07660329376 -2.9179

C 0.630468395609 5.72339670624 -2.9179

O -1.20335664569 1.90747484997 3.6202

O -1.20335664569 8.70747484997 3.6202

O 1.15772705266 -2.0168420765 3.527

O 1.15772705266 4.7831579235 3.527

C 0.00618352817951 -0.0321101849769 5.012

C 0.00618352817951 6.76788981502 5.012

O 1.81846961524 -3.14848176562 -1.3685

O 1.81846961524 3.65151823438 -1.3685

C 2.44805954988 -4.22836352035 -0.6654

C 2.44805954988 2.57163647965 -0.6654

C 3.00405307436 -5.18157255343 -1.7052

C 3.00405307436 1.61842744657 -1.7052

O -1.1243539857 2.04303822892 -3.5254

O -1.1243539857 8.84303822892 -3.5254

C 0.0185764681195 0.0204246133918 -5.0117

C 0.0185764681195 6.82042461339 -5.0117

O 1.12155551363 -1.94939113311 -3.6251

O 1.12155551363 4.85060886689 -3.6251

O -1.81680455173 3.15059779102 1.3703

O -1.81680455173 9.95059779102 1.3703

C -2.44854615242 4.22900634067 0.6664

C -2.44854615242 11.0290063407 0.6664

C -3.02069153163 5.17439120677 1.7049

C -3.02069153163 11.9743912068 1.7049

H 1.66061916883 -2.89447677243 1.3372

H 1.66061916883 3.90552322757 1.3372

H -1.64160417438 2.90834183594 -1.335

H -1.64160417438 9.70834183594 -1.335

H -3.2475898507 3.82599062356 0.0037

H -3.2475898507 10.6259906236 0.0037

H -1.70210559756 4.74333337483 0.0192

H -1.70210559756 11.5433333748 0.0192

H -3.51749678752 6.03048315144 1.2068

H -3.51749678752 12.8304831514 1.2068

H -3.76378208649 4.65426180242 2.3404

H -3.76378208649 11.4542618024 2.3404

H -2.21963514004 5.56552083682 2.3618

H -2.21963514004 12.3655208368 2.3618

H 1.70374194236 -4.73476760716 -0.0106

H 1.70374194236 2.06523239284 -0.0106

H 3.25570658026 -3.82984921155 -0.0107

H 3.25570658026 2.97015078845 -0.0107

H 3.50118153533 -6.03782430571 -1.2087

H 3.50118153533 0.76217569429 -1.2087

H 2.19405053035 -5.56920699294 -2.353

H 2.19405053035 1.23079300706 -2.353

H 3.74269843869 -4.66874385322 -2.3505

H 3.74269843869 2.13125614678 -2.3505

H -0.883360528953 -0.495574682453 -5.3951

H -0.883360528953 6.30442531755 -5.3951

H 0.0309866616846 1.06912952761 -5.3547

H 0.0309866616846 7.86912952761 -5.3547

H 0.916977885019 -0.510852286269 -5.3803

H 0.916977885019 6.28914771373 -5.3803

H -0.877563276514 -0.580215818221 5.3992

H -0.877563276514 6.21978418178 5.3992

H 0.92858769264 -0.530361062935 5.3557

H 0.92858769264 6.26963893707 5.3557

H -0.0267051782393 1.00845472554 5.3785

H -0.0267051782393 7.80845472554 5.3785

Organic linker trimers:

R=H

C -5.662942 0.251379 12.732385

C -4.929684 -0.013412 11.567487

C -5.601402 -0.264055 10.354616

C -7.003350 -0.241023 10.300684

C -7.745607 0.023848 11.469130

C -7.061760 0.260799 12.681174

N -3.470000 -0.007200 11.590000

C -2.867349 -1.283482 11.450135

C -1.411334 -1.280011 11.217141

C -0.710808 -0.032922 11.248111

C -1.405141 1.209891 11.214998

C -2.877670 1.247915 11.350775

C 0.712693 0.008508 11.281136

C 1.394761 -1.236922 11.427573

C 0.724620 -2.444559 11.311004

C	-0.676764	-2.488269	11.087335
C	1.418551	1.245477	11.155821
C	2.898111	1.207056	11.135194
N	3.470000	0.002799	11.610000
C	2.848225	-1.261449	11.689241
C	-0.720840	2.407613	11.047649
C	0.691720	2.448403	10.973902
C	4.926931	-0.025622	11.670497
C	5.654334	-0.460183	10.547835
C	7.051608	-0.460152	10.581987
C	7.743783	-0.063013	11.747191
C	7.007593	0.331573	12.882534
C	5.602893	0.363111	12.838254
C	9.240948	-0.032517	11.663980
O	9.910001	-0.007201	12.830001
O	3.486829	-2.272768	11.943253
O	3.625850	2.127447	10.772769
O	-1.183995	-3.698484	10.816224
C	-2.092122	-3.894152	9.697337
C	-3.037019	-5.028066	10.024919
O	1.247311	3.650317	10.687667
C	2.006649	4.353811	11.696267
C	2.917158	5.340306	10.997347
O	-3.557228	-2.291421	11.560554
O	-3.544973	2.272387	11.281371

C	-9.244534	0.033080	11.535028
O	-9.910000	0.022800	10.370000
O	-9.860000	0.052800	12.580000
O	9.860001	-0.027202	10.620001
C	5.720644	0.681241	7.164970
C	4.964885	0.089882	6.140036
C	5.599257	-0.529402	5.048702
C	6.994513	-0.544695	4.974922
C	7.763229	0.025693	6.008334
C	7.121332	0.629830	7.108190
N	3.520778	0.096249	6.185592
C	2.884108	-1.147465	6.407924
C	1.421270	-1.165686	6.132184
C	0.704620	0.046990	5.911268
C	1.378201	1.308265	5.948213
C	2.865949	1.331806	5.934506
C	-0.704819	-0.041324	5.673266
C	-1.378467	-1.302536	5.636517
C	-0.610588	-2.492156	5.751400
C	0.774144	-2.392948	6.056324
C	-1.421501	1.171387	5.452312
C	-0.774175	2.398623	5.527119
C	0.610485	2.497789	5.831910
C	-2.884544	1.153037	5.178186
N	-3.521247	-0.090221	5.403915

C	-2.866274	-1.325755	5.653274
C	9.251918	-0.002221	5.868513
O	9.910001	-0.007203	7.030001
O	3.519650	-2.134237	6.755445
O	3.534154	2.329558	5.683870
O	-1.085732	-3.744660	5.624535
C	-1.828167	-4.125861	4.434979
C	-2.798320	-5.227349	4.799183
O	1.086253	3.750438	5.957140
C	1.824372	4.133461	7.148774
C	2.793957	5.236131	6.786769
O	-3.534074	-2.323412	5.905531
C	-4.965219	-0.082819	5.453620
C	-5.724278	-0.671873	4.429774
C	-7.124700	-0.617479	4.489321
C	-7.763354	-0.012780	5.590871
C	-6.991345	0.554150	6.623735
C	-5.596202	0.535837	6.547287
O	-3.520487	2.139189	4.829614
C	-9.251877	0.020785	5.731657
O	-9.909999	0.022799	4.569998
O	-9.859999	0.052799	6.779998
O	9.860001	-0.027203	4.820001
C	5.600470	0.262616	1.245810
C	4.929638	0.010744	0.032591

C	5.663769	-0.250564	-1.132401
C	7.062795	-0.254451	-1.081247
C	7.745593	-0.015578	0.130894
C	7.002313	0.244919	1.299742
N	3.470002	0.002796	0.010000
C	2.877966	-1.253090	0.246544
C	1.405432	-1.215454	0.382691
C	0.711111	0.027477	0.352564
C	1.411255	1.274513	0.386638
C	2.867166	1.278758	0.153458
C	-0.712422	-0.013894	0.319124
C	-1.418587	-1.250787	0.442243
C	-0.691824	-2.454201	0.621113
C	0.720783	-2.413428	0.547269
C	-1.394299	1.231782	0.174451
C	-0.724475	2.439303	0.294027
C	0.676640	2.482470	0.519187
C	-2.847235	1.256748	-0.089179
N	-3.469998	-0.007203	-0.010001
C	-2.898269	-1.211451	0.464640
C	9.244492	-0.016035	0.065034
O	9.910002	-0.007204	1.230001
O	3.545597	-2.277478	0.313382
O	3.556840	2.287086	0.045614
O	-1.247331	-3.656830	0.904482

C	-2.010308	-4.356509	-0.103944
C	-2.922145	-5.342089	0.594640
O	1.183788	3.691846	0.794128
C	2.091138	3.883979	1.914328
C	3.037357	5.017870	1.590611
O	-3.625808	-2.131274	0.829052
C	-4.926935	0.023330	-0.069912
C	-5.604424	-0.369219	-1.235495
C	-7.009013	-0.332254	-1.280027
C	-7.743675	0.071688	-0.147015
C	-7.049939	0.471600	1.016343
C	-5.652698	0.466267	1.050570
O	-3.484793	2.268433	-0.344349
C	-9.240931	0.049651	-0.063932
O	-9.909998	0.022798	-1.230002
O	-9.859999	0.052798	0.979998
O	9.860002	-0.027205	-0.979999
H	7.661408	-0.440557	-1.984709
H	7.528700	0.446908	2.244571
H	1.291477	-3.350691	0.616274
H	-1.292178	3.379990	0.248489
H	-7.541104	-0.634729	-2.193748
H	-7.633136	0.785101	1.894793
H	1.453450	4.120312	2.795971
H	2.646223	2.951978	2.134902

H	2.476589	5.939063	1.336133
H	3.674269	5.229927	2.472586
H	3.684973	4.737396	0.739043
H	-2.600231	-3.637433	-0.707904
H	-1.287547	-4.868825	-0.779751
H	-3.651592	-4.794496	1.222011
H	-3.475273	-5.945968	-0.151776
H	-2.338698	-6.033323	1.235524
H	-10.855598	-0.011174	-0.970298
H	10.858713	0.004657	0.983062
H	7.510892	-1.011822	4.123624
H	7.726489	1.066649	7.916040
H	1.351137	-3.317911	6.199594
H	-1.350989	3.323617	5.383373
H	-7.732250	-1.051783	3.681947
H	-7.505047	1.021449	7.476564
H	1.072584	4.470265	7.896727
H	2.353462	3.262883	7.583903
H	2.263077	6.095201	6.330143
H	3.309500	5.591943	7.701045
H	3.550413	4.855294	6.074834
H	-2.357152	-3.254267	4.001813
H	-1.079039	-4.463353	3.684723
H	-3.552073	-4.845891	5.513601
H	-3.317116	-5.581782	3.886216

H	-2.267294	-6.087519	5.253552
H	-10.863195	0.022048	4.801477
H	10.863149	-0.001480	6.798273
H	7.636006	-0.766706	9.701883
H	7.538488	0.631554	13.797749
H	1.292131	-3.385305	11.357787
H	-1.291827	3.344498	10.976236
H	-7.530278	-0.441341	9.355816
H	-7.659733	0.450587	13.584289
H	1.281483	4.865518	12.369963
H	2.597400	3.637223	12.302389
H	2.333053	6.029231	10.354560
H	3.467942	5.946576	11.743556
H	3.648689	4.793388	10.371795
H	-2.648259	-2.963224	9.475015
H	-1.454917	-4.132010	8.815719
H	-3.683634	-4.746014	10.876750
H	-3.675143	-5.242798	9.144488
H	-2.475251	-5.948250	10.280831
H	-10.858741	0.018344	10.617110
H	10.855285	0.033399	12.570050
H	5.134736	-0.445819	-2.076064
H	5.019042	0.482083	2.154064
H	-5.027672	-0.701190	-2.111080
H	-5.120113	0.805074	1.951666

H	4.993563	-1.000440	4.260610
H	5.214159	1.173170	8.008693
H	-5.220484	-1.163905	3.584516
H	-4.988040	1.004400	7.334994
H	-5.021019	-0.485819	9.446233
H	-5.133165	0.445800	13.675818
H	5.122877	-0.795975	9.644918
H	5.024773	0.688421	13.715437

R=Me

C	7.052672	-0.404581	-1.031831
C	5.649771	-0.413414	-1.091883
C	4.935195	0.014137	0.048432
C	5.593718	0.425575	1.234429
C	7.003572	0.364806	1.271691
C	7.740332	-0.033980	0.141013
N	3.470000	0.002800	0.010000
C	2.878918	-1.230822	0.311118
C	1.404592	-1.199178	0.337446
C	0.709423	0.035852	0.188016
C	1.401143	1.286301	0.106347
C	2.860378	1.270818	-0.083747
C	0.729974	-2.400633	0.493805
C	-0.683523	-2.462484	0.477710
C	-1.409782	-1.257579	0.306509

C	-0.711624	-0.017796	0.143213
C	-1.396889	1.213800	-0.078081
C	-0.733199	2.429563	-0.104764
C	0.668724	2.500212	0.071576
C	-2.879856	-1.211827	0.415664
N	-3.470000	-0.007200	-0.010000
C	-2.856140	1.224938	-0.270191
C	-4.933507	0.010612	-0.047810
C	-5.594324	-0.485888	-1.195384
C	-7.002671	-0.433827	-1.233826
C	-7.737868	0.049862	-0.135501
C	-7.048263	0.521842	0.999607
C	-5.643143	0.549665	1.050696
O	1.185907	3.736930	0.148176
C	2.051900	4.097783	1.252734
O	-1.214603	-3.687309	0.667184
C	3.018977	5.163525	0.787202
C	-2.100106	-4.282597	-0.307792
C	-3.046801	-5.220291	0.410047
O	3.550452	2.254821	-0.332589
O	3.549237	-2.238625	0.508000
O	-3.590651	-2.123275	0.832353
O	-3.504696	2.210266	-0.600867
C	4.828019	0.971511	2.411868
C	4.922607	-0.866393	-2.330738

C	-4.805942	-1.054456	-2.347667
C	-4.926292	1.179772	2.214772
C	-9.237222	0.040462	-0.061840
O	-9.860000	0.052800	0.980000
O	-9.910000	0.022800	-1.230000
C	9.240047	-0.023181	0.064289
O	9.860000	-0.027200	-0.980000
O	9.910000	-0.007200	1.230000
H	7.645806	-0.691474	-1.913000
H	7.534104	0.659137	2.190663
H	1.308467	-3.327749	0.612823
H	-1.310608	3.355839	-0.235262
H	-7.534507	-0.795583	-2.126706
H	-7.635895	0.886685	1.855855
H	1.385772	4.461515	2.066785
H	2.585704	3.208048	1.640153
H	2.474845	6.039642	0.381741
H	3.642043	5.503575	1.638324
H	3.680602	4.751237	0.002955
H	-2.658090	-3.500232	-0.860396
H	-1.457375	-4.826877	-1.036586
H	-3.699302	-4.641756	1.090360
H	-3.678680	-5.758550	-0.324370
H	-2.481740	-5.970957	0.997588
H	-10.853849	-0.003182	-0.962951

H	10.855965	0.022823	0.972420
H	4.560902	2.034965	2.232268
H	5.417895	0.938171	3.346235
H	3.876678	0.432656	2.589076
H	4.158502	-0.127201	-2.646852
H	4.397183	-1.826455	-2.142220
H	5.623970	-1.021282	-3.171897
H	-4.018939	-0.351670	-2.689617
H	-5.461395	-1.282182	-3.209070
H	-4.297413	-1.997766	-2.053177
H	-4.625060	2.216970	1.957215
H	-3.998825	0.639509	2.483058
H	-5.561125	1.232400	3.118211
C	7.043705	-0.746441	5.787731
C	5.641896	-0.791947	5.815617
C	4.986720	-0.047948	6.824131
C	5.677261	0.673603	7.822658
C	7.083644	0.605884	7.817977
C	7.762767	-0.070876	6.790029
N	3.539499	-0.011557	6.812608
C	2.861188	-1.050287	7.474848
C	1.378444	-1.024851	7.346040
C	0.714753	0.028664	6.650292
C	1.459522	1.110464	6.078807
C	2.944702	1.039772	6.072406

C	0.655450	-2.078326	7.891068
C	-0.756152	-2.165976	7.770744
C	-1.458718	-1.114556	7.123140
C	-0.713811	-0.032845	6.551435
C	-1.377547	1.020822	5.855862
C	-0.654766	2.074934	5.311842
C	0.756964	2.162740	5.432264
C	-2.944303	-1.045835	7.127139
N	-3.538756	0.006952	6.388111
C	-2.860236	1.046205	5.727704
C	-4.986173	0.045388	6.377268
C	-5.679468	-0.672163	5.377548
C	-7.085666	-0.598997	5.382343
C	-7.762335	0.079163	6.410957
C	-7.040527	0.749744	7.414655
C	-5.638674	0.789608	7.387402
O	1.316692	3.257651	4.874553
C	2.098941	4.166950	5.687090
O	-1.316773	-3.259643	8.330683
C	3.087642	4.888856	4.798511
C	-2.093021	-4.172990	7.516842
C	-3.075918	-4.902130	8.406187
O	3.667977	1.819859	5.457840
O	3.469041	-1.919260	8.089744
O	-3.668008	-1.827443	7.738893

O	-3.468082	1.915700	5.112743
C	4.948974	1.530957	8.826942
C	4.869127	-1.623616	4.824728
C	-4.954534	-1.529879	4.371394
C	-4.862760	1.615993	8.380238
C	-9.250919	0.051427	6.532467
O	-9.860000	0.052800	7.580000
O	-9.910000	0.022800	5.370000
C	9.251081	-0.034662	6.667435
O	9.860000	-0.027200	5.620000
O	9.910000	-0.007200	7.830000
H	7.600803	-1.253469	4.985431
H	7.658880	1.119991	8.602642
H	1.193735	-2.885527	8.408552
H	-1.193163	2.882574	4.795307
H	-7.662804	-1.109561	4.596709
H	-7.595334	1.258052	8.217753
H	1.381336	4.873221	6.162671
H	2.623281	3.615317	6.491593
H	2.568234	5.461760	4.005086
H	3.683752	5.602181	5.401364
H	3.775364	4.157780	4.332275
H	-2.622296	-3.623185	6.714595
H	-1.371404	-4.874617	7.040195
H	-3.768139	-4.175483	8.872665

H	-3.667643	-5.620142	7.804297
H	-2.551558	-5.471333	9.198960
H	-10.859952	-0.027529	5.611235
H	10.859702	0.050470	7.589123
H	4.777407	2.546083	8.407180
H	5.526422	1.651672	9.763200
H	3.957056	1.122679	9.097214
H	3.955129	-1.112215	4.465777
H	4.544605	-2.574922	5.298035
H	5.483609	-1.877325	3.940395
H	-3.960876	-1.124856	4.102650
H	-5.532620	-1.645757	3.434911
H	-4.787559	-2.546865	4.788453
H	-4.534814	2.567467	7.909630
H	-3.950918	1.100065	8.738004
H	-5.475766	1.869732	9.265703
C	7.049980	-0.514842	12.200358
C	5.644937	-0.548733	12.149627
C	4.933812	-0.013228	13.248834
C	5.592277	0.483384	14.397578
C	7.000916	0.436782	14.435708
C	7.737883	-0.041395	13.336037
N	3.470000	0.002800	13.210000
C	2.856349	-1.228024	13.476534
C	1.396910	-1.218323	13.282870

C	0.711555	0.011886	13.053377
C	1.410033	1.250276	12.880001
C	2.880354	1.203568	12.772655
C	0.732965	-2.433968	13.315536
C	-0.668792	-2.505116	13.137149
C	-1.401351	-1.291450	13.097041
C	-0.709477	-0.041827	13.008523
C	-1.404412	1.192286	12.852092
C	-0.729762	2.392303	12.685231
C	0.683825	2.453812	12.698030
C	-2.860934	-1.275350	13.285493
N	-3.470000	-0.007200	13.190000
C	-2.878806	1.224369	12.881049
C	-4.935203	-0.016861	13.152280
C	-5.595391	-0.429583	11.967796
C	-7.005144	-0.363659	11.930205
C	-7.740280	0.041880	13.059596
C	-7.051297	0.412941	14.231738
C	-5.648394	0.416556	14.291389
O	1.214668	3.676277	12.494247
C	2.101384	4.284221	13.460627
O	-1.186439	-3.741958	13.063905
C	3.045519	5.213904	12.729042
C	-2.048992	-4.105207	11.957243
C	-3.015439	-5.171983	12.421319

O	3.591374	2.111442	12.348699
O	3.504710	-2.210965	13.814504
O	-3.551733	-2.258630	13.535318
O	-3.549142	2.231485	12.679557
C	4.800765	1.044832	15.551064
C	4.929657	-1.180480	10.985493
C	-4.832078	-0.982263	10.791626
C	-4.919352	0.870116	15.529104
C	-9.240050	0.039909	13.135719
O	-9.860000	0.052800	14.180000
O	-9.910000	0.022800	11.970000
C	9.237238	-0.023484	13.261888
O	9.860000	-0.027200	12.220000
O	9.910000	-0.007200	14.430000
H	7.639087	-0.875098	11.343257
H	7.531489	0.799500	15.328831
H	1.310030	-3.359613	13.451382
H	-1.308340	3.318405	12.559460
H	-7.536585	-0.657873	11.011847
H	-7.643155	0.705949	15.111716
H	1.459213	4.836682	14.183828
H	2.661102	3.509342	14.021715
H	2.478660	5.956411	12.132869
H	3.678043	5.762580	13.455253
H	3.697510	4.627480	12.055148

H	-2.583393	-3.216874	11.567646
H	-1.380058	-4.468699	11.145281
H	-3.679644	-4.759882	13.203658
H	-3.635541	-5.513628	11.568673
H	-2.471313	-6.046943	12.829023
H	-10.856111	-0.000223	12.227398
H	10.853713	0.025397	14.163007
H	4.273885	1.977628	15.255457
H	5.456704	1.288208	16.407931
H	4.028592	0.329509	15.900828
H	4.002307	-0.641462	10.714688
H	4.628390	-2.217500	11.244260
H	5.565580	-1.234443	10.082802
H	-3.877422	-0.449732	10.613574
H	-5.420917	-0.946862	9.856630
H	-4.571720	-2.046936	10.973796
H	-4.386524	1.825474	15.337700
H	-4.160900	0.126772	15.849072
H	-5.620436	1.033547	16.368955

R=Et

C	7.014548	-0.382053	-1.119159
C	5.610222	-0.403582	-1.128149
C	4.930816	0.028400	0.033185
C	5.633140	0.467583	1.186372

C	7.043086	0.409634	1.173428
C	7.742589	0.002809	0.023180
N	3.462800	0.013100	0.040100
C	2.883690	-1.132726	0.598779
C	1.409199	-1.108120	0.612925
C	0.709568	0.082824	0.264361
C	1.397366	1.308584	-0.006159
C	2.854293	1.262094	-0.209932
C	0.738664	-2.276675	0.942956
C	-0.673512	-2.354364	0.903220
C	-1.405113	-1.195374	0.537646
C	-0.710284	0.012724	0.208710
C	-1.396867	1.193240	-0.202926
C	-0.739122	2.396060	-0.400670
C	0.660834	2.501379	-0.224647
C	-2.879417	-1.154902	0.589916
N	-3.467200	-0.016900	0.010100
C	-2.851331	1.165509	-0.419252
C	-4.932045	-0.001507	-0.047467
C	-5.582122	-0.515397	-1.194276
C	-6.990240	-0.459182	-1.252114
C	-7.739973	0.033850	-0.170412
C	-7.062678	0.522602	0.964863
C	-5.658685	0.558137	1.032505
O	1.167083	3.739637	-0.333578

C	2.038937	4.270113	0.695475
O	-1.202195	-3.544853	1.255298
C	3.004655	5.249767	0.067113
C	-2.021103	-4.292259	0.328114
C	-3.014972	-5.121608	1.112830
O	3.540496	2.201657	-0.601395
O	3.560769	-2.076285	0.996424
O	-3.594170	-2.024940	1.084452
O	-3.496776	2.087893	-0.903416
C	4.941308	1.077820	2.387606
C	4.869310	-0.912994	-2.345532
C	-4.817151	-1.177263	-2.320824
C	-4.987312	1.231227	2.210537
C	-9.239774	0.002718	-0.110132
O	-9.867200	-0.006900	0.930100
O	-9.897200	-0.016900	-1.289900
C	9.237886	0.032748	-0.110031
O	9.812800	0.033100	-1.179900
O	9.952800	0.063100	1.030100
H	7.576784	-0.669270	-2.021126
H	7.605866	0.720188	2.068465
H	1.319509	-3.169808	1.212996
H	-1.318691	3.289064	-0.674016
H	-7.510473	-0.835633	-2.146459
H	-7.660230	0.894311	1.812169

H	1.377289	4.764678	1.442232
H	2.574782	3.451153	1.212465
H	3.656327	4.723777	-0.654977
H	2.459625	6.059896	-0.457314
H	3.637197	5.708117	0.853709
H	-2.537238	-3.608112	-0.374031
H	-1.332504	-4.935294	-0.267106
H	-2.493008	-5.792549	1.824351
H	-3.696780	-4.455342	1.673897
H	-3.614211	-5.747794	0.422150
H	-10.844596	-0.060673	-1.037999
H	10.887533	0.102955	0.735640
H	5.379566	0.664330	3.317534
H	3.868515	0.798495	2.410560
C	5.080173	2.608998	2.432493
H	3.865735	-0.443946	-2.399744
C	4.730940	-2.445281	-2.351395
H	5.414666	-0.584592	-3.254763
C	-4.866557	-2.712324	-2.228007
H	-3.763133	-0.834786	-2.318492
H	-5.246396	-0.847969	-3.290396
H	-3.956692	0.846285	2.344829
H	-5.530258	0.966675	3.139700
C	-4.950924	2.764627	2.090846
H	-5.978315	3.180065	2.048058

H	-4.423355	3.088929	1.172488
H	-4.440918	3.191494	2.976666
H	-5.906105	-3.084027	-2.331826
H	-4.495120	-3.059578	-1.242960
H	-4.254992	-3.183896	-3.024132
H	4.158032	-2.790040	-3.236208
H	4.219200	-2.805654	-1.436567
H	5.727685	-2.930819	-2.380090
H	4.718264	3.071426	1.493942
H	6.140892	2.905643	2.559695
H	4.505474	3.012526	3.289124
C	7.009381	-0.687841	5.690639
C	5.608498	-0.738166	5.766106
C	4.987826	-0.017334	6.816561
C	5.719603	0.674755	7.810713
C	7.124577	0.612086	7.748226
C	7.766590	-0.032023	6.676559
N	3.539714	0.033014	6.844403
C	2.863989	-1.025501	7.476614
C	1.386863	-1.034803	7.302749
C	0.715789	0.045530	6.657437
C	1.448924	1.176044	6.175505
C	2.934219	1.121284	6.167027
C	0.677567	-2.144388	7.745342
C	-0.727116	-2.257206	7.570624

C	-1.439787	-1.169572	6.999714
C	-0.706829	-0.038026	6.519194
C	-1.378145	1.040672	5.872132
C	-0.669040	2.148361	5.425559
C	0.735636	2.260820	5.598310
C	-2.924526	-1.114015	7.009436
N	-3.530823	-0.030459	6.324701
C	-2.855124	1.027582	5.692839
C	-4.980446	0.005158	6.323164
C	-5.680193	-0.699405	5.315442
C	-7.086325	-0.628472	5.328156
C	-7.762973	0.032802	6.367777
C	-7.036780	0.687204	7.378015
C	-5.633683	0.730589	7.349352
O	1.282165	3.412413	5.155472
C	2.015885	4.255756	6.076826
O	-1.273690	-3.412329	8.002216
C	3.006858	5.089998	5.296906
C	-2.010246	-4.244928	7.073088
C	-3.019614	-5.071692	7.838579
O	3.646953	1.942157	5.594614
O	3.473614	-1.896242	8.090057
O	-3.637728	-1.930951	7.586315
O	-3.462270	1.894437	5.071226
C	5.051303	1.521736	8.875228

C	4.830508	-1.577998	4.772653
C	-4.981688	-1.566401	4.288246
C	-4.887266	1.572467	8.365122
C	-9.252188	0.002555	6.484845
O	-9.867200	-0.006900	7.530100
O	-9.897200	-0.016900	5.310100
C	9.247658	0.021746	6.492654
O	9.812800	0.033100	5.420100
O	9.952800	0.063100	7.630100
H	7.536687	-1.175620	4.856087
H	7.728861	1.107343	8.523488
H	1.219496	-2.976761	8.217200
H	-1.210176	2.978305	4.949623
H	-7.664558	-1.128803	4.536567
H	-7.589311	1.182329	8.191223
H	1.265816	4.893466	6.597175
H	2.528937	3.640243	6.842197
H	3.758484	4.438825	4.813052
H	2.493421	5.688647	4.517885
H	3.531082	5.786744	5.980535
H	-2.507835	-3.621901	6.303716
H	-1.262321	-4.889912	6.558430
H	-2.524319	-5.667699	8.630686
H	-3.780142	-4.415919	8.302650
H	-3.531515	-5.770593	7.147742

H	-10.850674	-0.071137	5.536052
H	10.891332	0.127480	7.350735
H	5.542386	1.341389	9.853506
H	3.996345	1.211297	9.011866
C	5.110848	3.025533	8.558320
H	3.797139	-1.190099	4.660320
C	4.787098	-3.067394	5.157591
H	5.294821	-1.484327	3.769595
C	-5.063959	-3.063584	4.631512
H	-3.920289	-1.265675	4.183372
H	-5.435704	-1.402438	3.289343
H	-3.856407	1.189207	8.504943
H	-5.381576	1.473882	9.353856
C	-4.840028	3.062384	7.982661
H	-5.863985	3.482755	7.916337
H	-4.354747	3.212994	6.998269
H	-4.284506	3.639531	8.748894
H	-6.115328	-3.416237	4.622609
H	-4.659918	-3.265661	5.643467
H	-4.496922	-3.663024	3.890939
H	4.210341	-3.641954	4.405137
H	4.326233	-3.215983	6.154129
H	5.810305	-3.492276	5.197829
H	4.663552	3.243954	7.567908
H	6.159473	3.385230	8.529087

H	4.571770	3.606469	9.333555
C	7.012582	-0.529736	12.125278
C	5.606416	-0.571589	12.124823
C	4.929797	-0.011258	13.236799
C	5.631193	0.507186	14.350262
C	7.041140	0.459155	14.340089
C	7.741501	-0.029666	13.223781
N	3.462800	0.013100	13.240100
C	2.855623	-1.150468	13.724495
C	1.395509	-1.169449	13.555241
C	0.706944	0.008445	13.140726
C	1.399154	1.217744	12.806531
C	2.867715	1.163869	12.697328
C	0.737721	-2.365304	13.790397
C	-0.662704	-2.472847	13.631216
C	-1.398496	-1.288055	13.369381
C	-0.711608	-0.063058	13.086293
C	-1.408284	1.126929	12.724360
C	-0.742176	2.302519	12.416257
C	0.668575	2.385144	12.469904
C	-2.859946	-1.248710	13.524907
N	-3.467200	-0.016900	13.210100
C	-2.880632	1.143357	12.696355
C	-4.934573	-0.039184	13.154810
C	-5.580341	-0.472563	11.968346

C	-6.989702	-0.407913	11.912077
C	-7.741814	0.004548	13.026968
C	-7.068510	0.382047	14.204634
C	-5.665825	0.394173	14.282723
O	1.191687	3.587199	12.155529
C	2.061825	4.281637	13.078256
O	-1.172426	-3.705646	13.792510
C	3.033553	5.132207	12.289068
C	-2.011671	-4.293541	12.767804
C	-3.013401	-5.222874	13.417686
O	3.571751	2.028450	12.178050
O	3.511310	-2.067847	14.204906
O	-3.555145	-2.180753	13.917765
O	-3.552405	2.088675	12.293305
C	4.921065	1.166068	15.514009
C	4.879974	-1.249056	10.982858
C	-4.826121	-1.079463	10.803454
C	-4.983911	0.901353	15.534618
C	-9.242573	-0.006525	13.087608
O	-9.867200	-0.006900	14.130100
O	-9.897200	-0.016900	11.910100
C	9.235065	0.021958	13.087726
O	9.812800	0.033100	12.020100
O	9.952800	0.063100	14.230100
H	7.571107	-0.901497	11.251950

H	7.601909	0.842006	15.206651
H	1.319578	-3.252973	14.076617
H	-1.325638	3.195229	12.149723
H	-7.508744	-0.715385	10.990392
H	-7.672293	0.673491	15.077449
H	1.407280	4.906015	13.728141
H	2.596114	3.561392	13.727805
H	3.678843	4.483720	11.667129
H	2.491333	5.843836	11.634478
H	3.672899	5.715738	12.981338
H	-2.516971	-3.503321	12.179238
H	-1.328755	-4.843593	12.081288
H	-2.499100	-6.004559	14.011974
H	-3.686206	-4.646788	14.079169
H	-3.619792	-5.723299	12.636268
H	-10.847374	-0.047821	12.151695
H	10.884371	0.120673	13.927238
H	5.372650	0.806065	16.462151
H	3.859071	0.850092	15.541598
C	5.006284	2.700680	15.449474
H	3.855757	-0.838190	10.875492
C	4.809501	-2.778738	11.135908
H	5.396869	-1.019820	10.030421
C	-4.918187	-2.614615	10.777716
H	-3.761823	-0.769171	10.820883

H	-5.237414	-0.691924	9.850307
H	-3.976621	0.446645	15.626139
H	-5.561187	0.555196	16.417385
C	-4.870640	2.435171	15.561321
H	-5.876313	2.903399	15.551898
H	-4.326264	2.813633	14.673420
H	-4.342519	2.780100	16.473519
H	-5.960893	-2.944265	10.595215
H	-4.598744	-3.051489	11.743780
H	-4.283053	-3.015987	9.963720
H	4.267375	-3.212501	10.272698
H	4.301984	-3.074572	12.074671
H	5.828539	-3.215923	11.158176
H	4.611592	3.075837	14.483746
H	6.057204	3.045574	15.526223
H	4.432515	3.171089	16.274045

R=iPr

O	-3.465455	-1.465898	1.647855
C	-2.776223	-0.757516	0.926177
C	-1.308948	-0.656855	0.984521
C	-0.631847	0.114729	-0.010429
C	-1.330027	0.880207	-0.985563
C	-2.799640	0.848041	-1.040288
N	-3.390060	0.015630	-0.089690

C	0.788397	0.130677	-0.093600
C	1.482638	-0.720806	0.807345
C	0.842789	-1.425822	1.812046
C	-0.564404	-1.376993	1.957278
C	1.471471	0.925437	-1.063295
C	2.943695	0.912845	-1.085540
N	3.540280	0.020380	-0.165050
C	2.932642	-0.904877	0.673845
C	-0.679171	1.658075	-1.932584
C	0.735018	1.712268	-1.986415
C	5.009843	0.027811	-0.124133
C	5.716963	-0.843037	-0.989094
C	7.121693	-0.847861	-0.932422
C	7.823378	-0.071292	0.007968
C	7.097030	0.766911	0.864575
C	5.691090	0.868328	0.792098
C	4.999551	-1.737267	-1.993394
C	5.306991	-3.223851	-1.742804
C	5.055632	1.948578	1.661633
C	4.994309	3.281793	0.886415
C	9.322717	-0.043046	-0.068740
O	10.000700	-0.011800	1.095900
O	3.560767	-1.776917	1.270511
O	3.656785	1.594395	-1.812887
O	-1.195011	-2.013081	2.955836

C	-0.406817	-2.731810	3.925622
C	-1.352435	-3.403217	4.894790
O	1.399466	2.456635	-2.877094
C	0.685405	3.234300	-3.851485
C	1.719712	3.931718	-4.712269
C	-4.857707	-0.021830	-0.142785
C	-5.485345	-0.772920	-1.164887
C	-6.893278	-0.732256	-1.265682
C	-7.662607	-0.032102	-0.323929
C	-7.009348	0.667756	0.708263
C	-5.610027	0.734467	0.797279
O	-3.461842	1.478945	-1.861544
C	-4.690234	-1.632646	-2.141384
C	-5.024435	-3.123922	-1.953446
C	-9.161985	-0.061862	-0.260011
O	-9.829300	-0.071800	-1.434100
C	-4.949207	1.615516	1.854055
C	-4.429309	2.929011	1.231547
C	3.713022	1.622447	2.315979
C	5.313636	-1.303745	-3.436809
C	-4.881705	-1.167192	-3.595132
C	-5.859600	1.932007	3.048242
O	-9.779300	-0.061800	0.785900
O	9.920700	-0.031800	-1.124100
H	7.697961	-1.471238	-1.632217

H	7.636367	1.391193	1.594099
H	1.481142	-2.040727	2.458259
H	-1.318197	2.211509	-2.633448
H	-7.398516	-1.287613	-2.070229
H	-7.632872	1.177762	1.453585
H	0.035803	2.564449	-4.459953
H	0.024738	3.966317	-3.334311
H	2.373260	3.190782	-5.212733
H	1.219357	4.544731	-5.486760
H	2.360524	4.592039	-4.095522
H	0.227736	-3.484861	3.408257
H	0.270001	-2.016138	4.446370
H	-0.774722	-3.930708	5.678740
H	-2.006853	-2.661949	5.387396
H	-1.998647	-4.135627	4.375138
H	-10.774472	-0.119100	-1.172770
H	10.944046	0.053005	0.832023
H	-3.614769	-1.513804	-1.897224
H	-4.612159	-0.097689	-3.698906
H	-5.933705	-1.291153	-3.926742
H	-4.244786	-1.760803	-4.282564
H	-6.086245	-3.335889	-2.197941
H	-4.850602	-3.439597	-0.904780
H	-4.396188	-3.753857	-2.616115
H	-4.083003	1.057179	2.263056

H	-6.711199	2.582077	2.758048
H	-5.278962	2.462426	3.826123
H	-6.262018	1.014721	3.519806
H	-3.857953	3.508693	1.986806
H	-5.278364	3.558610	0.892547
H	-3.780581	2.767766	0.350697
H	5.770152	2.096283	2.500445
H	3.908967	-1.603526	-1.844580
H	2.862402	1.689897	1.611406
H	3.519603	2.332420	3.136387
H	3.710027	0.610454	2.765045
H	5.995069	3.574036	0.509761
H	4.625448	4.094632	1.545684
H	4.324462	3.204513	0.006372
H	6.391627	-1.424328	-3.671092
H	5.048352	-0.238688	-3.591302
H	4.741836	-1.917764	-4.162818
H	6.382568	-3.449267	-1.896131
H	4.729532	-3.865054	-2.440296
H	5.041575	-3.509272	-0.705340
O	-3.438450	-1.767974	8.311751
C	-2.790185	-1.082479	7.532366
C	-1.312521	-0.931117	7.594114
C	-0.645789	-0.150868	6.602180
C	-1.375116	0.493938	5.569883

C	-2.845219	0.338371	5.456275
N	-3.466219	-0.389534	6.482662
C	0.775073	0.017164	6.592339
C	1.516653	-0.750990	7.525678
C	0.883658	-1.500640	8.505622
C	-0.533583	-1.554737	8.610539
C	1.436836	0.907234	5.693865
C	2.906227	1.113808	5.848551
N	3.602424	0.133348	6.617527
C	2.993374	-0.813737	7.447651
C	-0.740230	1.325129	4.656617
C	0.650654	1.612649	4.738468
C	5.056005	0.172375	6.657956
C	5.756405	-0.636154	5.722227
C	7.153475	-0.658115	5.806089
C	7.840563	0.036788	6.813746
C	7.126027	0.820282	7.725641
C	5.721088	0.936990	7.647552
C	5.059517	-1.445113	4.630042
C	4.596183	-2.831260	5.124732
C	5.087745	1.949860	8.602632
C	5.191337	3.366007	7.994819
C	9.329650	-0.001534	6.733955
O	10.000700	-0.011800	7.895900
O	3.638340	-1.640835	8.088335

O	3.529210	2.048573	5.363061
O	-1.134206	-2.191754	9.627957
C	-0.316215	-2.747230	10.676942
C	-1.228501	-3.352594	11.719219
O	1.225767	2.520647	3.936382
C	0.428544	3.202360	2.948259
C	1.268018	4.293925	2.314672
C	-4.920263	-0.363354	6.495595
C	-5.640946	-1.132793	5.547569
C	-7.037063	-0.969114	5.502060
C	-7.691967	-0.161307	6.439484
C	-6.958443	0.534214	7.410737
C	-5.556945	0.499515	7.428209
O	-3.486564	0.849925	4.544281
C	-4.977225	-2.184486	4.666839
C	-4.811091	-3.478285	5.493994
C	-9.175626	-0.094749	6.535612
O	-9.829300	-0.071800	5.365900
C	-4.787702	1.388515	8.406366
C	-4.170325	2.606255	7.685510
C	3.671501	1.687460	9.122679
C	5.940414	-1.614037	3.382988
C	-5.738765	-2.475465	3.366206
C	-5.644100	1.871195	9.585789
O	-9.779300	-0.061800	7.585900

O	9.920700	-0.031800	5.675900
H	7.747173	-1.218743	5.073419
H	7.668800	1.380723	8.502724
H	1.535806	-2.059909	9.188183
H	-1.379423	1.780623	3.890089
H	-7.632174	-1.508944	4.754027
H	-7.514407	1.121712	8.151556
H	0.077231	2.464433	2.191021
H	-0.470668	3.639940	3.436989
H	2.129980	3.879105	1.760940
H	0.642308	4.869919	1.604434
H	1.651477	4.988932	3.086714
H	0.365054	-3.517032	10.250368
H	0.315716	-1.941120	11.115966
H	-0.621899	-3.802309	12.529711
H	-1.884208	-2.584085	12.167507
H	-1.868245	-4.139787	11.276201
H	-10.781740	-0.068964	5.603159
H	10.948930	-0.005664	7.642135
H	-3.974081	-1.815770	4.368769
H	-5.928760	-1.554575	2.782613
H	-6.707209	-2.982356	3.558473
H	-5.132329	-3.139943	2.722830
H	-5.806030	-3.866316	5.795819
H	-4.231187	-3.312218	6.423411

H	-4.309513	-4.265886	4.895688
H	-3.959517	0.786903	8.839440
H	-6.446820	2.560882	9.252591
H	-5.010017	2.408956	10.312015
H	-6.113516	1.029655	10.132467
H	-3.578729	3.213138	8.401647
H	-4.970368	3.253438	7.269978
H	-3.508198	2.328457	6.843871
H	5.740001	1.933673	9.503442
H	4.152174	-0.877852	4.326524
H	2.884874	1.901504	8.372071
H	3.483927	2.336276	9.994030
H	3.546450	0.646719	9.478580
H	6.235923	3.612994	7.718521
H	4.842375	4.124587	8.725523
H	4.575653	3.440820	7.076203
H	6.769200	-2.328546	3.564768
H	6.388716	-0.655887	3.059826
H	5.338219	-1.998583	2.540695
H	5.474273	-3.446753	5.411123
H	4.063672	-3.360228	4.307364
H	3.935788	-2.781519	6.009192
O	-3.479602	-1.323424	15.358143
C	-2.782880	-0.774320	14.514302
C	-1.316311	-0.861207	14.413245

C	-0.634408	0.008272	13.510130
C	-1.329233	0.880857	12.630586
C	-2.796548	0.853570	12.572208
N	-3.390060	0.015630	13.510310
C	0.785900	0.041674	13.444941
C	1.476755	-0.914375	14.240053
C	0.822693	-1.805256	15.079407
C	-0.587033	-1.780611	15.209214
C	1.468492	1.001150	12.633300
C	2.937051	1.072240	12.715673
N	3.540280	0.020380	13.434950
C	2.944002	-0.978287	14.204829
C	-0.679115	1.775106	11.795762
C	0.730372	1.901805	11.819973
C	5.010853	0.033645	13.506188
C	5.734244	-0.668227	12.503163
C	7.135828	-0.652980	12.574369
C	7.823330	-0.035751	13.630812
C	7.083408	0.613055	14.629759
C	5.672448	0.697158	14.574386
C	5.060727	-1.402362	11.343256
C	4.493275	-2.777930	11.752104
C	5.027316	1.606742	15.629685
C	4.927792	3.051075	15.093575
C	9.319504	-0.023781	13.532147

O	10.000700	-0.011800	14.695900
O	3.602959	-1.810579	14.822879
O	3.639887	1.953127	12.231843
O	-1.257429	-2.598385	16.028365
C	-0.550349	-3.524556	16.868641
C	-1.585141	-4.246053	17.709223
O	1.374346	2.832112	11.102563
C	0.625767	3.725599	10.257002
C	1.585618	4.744884	9.676979
C	-4.861679	-0.007433	13.453000
C	-5.518078	-0.874100	12.538930
C	-6.920471	-0.783583	12.433487
C	-7.668443	0.054336	13.275367
C	-6.993411	0.858870	14.209893
C	-5.587944	0.879700	14.281507
O	-3.453556	1.523844	11.779262
C	-4.786311	-1.959743	11.757621
C	-4.625687	-3.201316	12.664963
C	-9.167919	-0.021145	13.338694
O	-9.829300	-0.071800	12.165900
C	-4.891560	1.855884	15.222049
C	-5.056116	3.300453	14.712384
C	3.707035	1.158422	16.276520
C	6.001199	-1.588028	10.144177
C	-5.479941	-2.360482	10.448734

C	-5.358607	1.689718	16.677088
O	-9.779300	-0.061800	14.385900
O	9.920700	-0.031800	12.475900
H	7.735662	-1.125319	11.787001
H	7.612617	1.110580	15.457285
H	1.456355	-2.505046	15.639806
H	-1.317310	2.388793	11.146897
H	-7.451858	-1.417049	11.710874
H	-7.587533	1.487677	14.889600
H	0.123447	3.135728	9.456041
H	-0.166508	4.226116	10.857098
H	2.347586	4.264309	9.035406
H	1.023300	5.476267	9.064055
H	2.107245	5.292394	10.486623
H	0.029566	-4.235686	16.237000
H	0.174918	-2.969677	17.505525
H	-1.088950	-4.967827	18.386589
H	-2.161005	-3.523348	18.319663
H	-2.298487	-4.796529	17.065580
H	-10.772816	-0.174194	12.416813
H	10.943890	0.025045	14.426244
H	-3.777280	-1.578390	11.488280
H	-5.673565	-1.491850	9.790867
H	-6.441778	-2.880157	10.638203
H	-4.831489	-3.050602	9.879445

H	-5.626324	-3.584061	12.953820
H	-4.080305	-2.983206	13.603117
H	-4.098263	-4.013530	12.125637
H	-3.806756	1.626268	15.203705
H	-6.432824	1.939908	16.796883
H	-4.785908	2.362398	17.347775
H	-5.208426	0.645855	17.018211
H	-4.497025	4.011732	15.355017
H	-6.122858	3.607073	14.717098
H	-4.683039	3.397354	13.673073
H	5.769908	1.627888	16.457225
H	4.206980	-0.772421	11.008154
H	2.831842	1.308112	15.612961
H	3.519699	1.770640	17.181809
H	3.736850	0.095494	16.582926
H	5.917613	3.419766	14.758188
H	4.554892	3.733555	15.885141
H	4.245737	3.112377	14.223051
H	6.789084	-2.338502	10.360579
H	6.503867	-0.645021	9.859006
H	5.430762	-1.934704	9.265783
H	5.316557	-3.452367	12.067051
H	3.987946	-3.242125	10.880397
H	3.779787	-2.730782	12.593775
